# Plasma lipidomic and metabolomic profiles in high‐grade glioma patients before and after 72‐h presurgery water‐only fasting

**DOI:** 10.1002/1878-0261.70003

**Published:** 2025-02-24

**Authors:** Iris Divé, Lisa Hahnefeld, Katharina J. Wenger, Donat Kögel, Joachim Steinbach, Gerd Geisslinger, Michael W. Ronellenfitsch, Irmgard Tegeder

**Affiliations:** ^1^ Dr. Senckenberg Institute of Neurooncology Goethe University Frankfurt, University Hospital Germany; ^2^ Center for Personalized Translational Epilepsy Research (CePTER) Goethe‐University Frankfurt, University Hospital Germany; ^3^ University Cancer Center Frankfurt (UCT) Goethe University Frankfurt, University Hospital Germany; ^4^ German Cancer Consortium (DKTK), Partner Site Frankfurt/Mainz a partnership between DKFZ and University Hospital Frankfurt Germany; ^5^ Institute for Clinical Pharmacology, Faculty of Medicine Goethe University Frankfurt Germany; ^6^ Fraunhofer Institute for Translational Medicine and Pharmacology ITMP and Fraunhofer Cluster of Excellence for Immune Mediated Diseases CIMD Frankfurt am Main Germany; ^7^ Institute of Neuroradiology Goethe‐University Frankfurt, University Hospital Germany; ^8^ Frankfurt Cancer Institute (FCI) Goethe University Frankfurt, University Hospital Germany; ^9^ Department of Neurosurgery, Experimental Neurosurgery Goethe‐University Frankfurt, Neuroscience Center Germany

**Keywords:** fasting, glioblastoma, lipidomic, lysophosphatidylcholines, metabolomic, plasma

## Abstract

Glioblastoma (GB) is the most aggressive primary brain tumor with poor prognosis despite multimodal therapy. Calorie‐restricted diets have emerged as putative strategies to augment anticancer therapies. We employed UHPLC‐high‐resolution mass spectrometry analyses of plasma lipids and polar metabolites to assess the systemic metabolic effects of a 72‐h preoperative fasting period in IDH‐wild‐type glioma patients (*n* = 9 GB and *n* = 1 diffuse pediatric‐type high‐grade H3/IDH‐wildtype) who participated in the prospective ERGO3 trial (NCT04461938). Fasting reduced lysophosphatidylcholines (LPC, LPC‐O), lysophosphatidylethanolamines (LPE, LPE‐O), and increased free fatty acids and carnitines. Triglyceride (TG) profiles shifted from short‐chain TGs (42–48 C‐atoms) to very long‐chain TGs (58–60 C‐atoms) indicating an exploitation of neutral lipid stores. Branched‐chain amino acids, aminobutyric acid, and uric acids were increased, and glucose reduced after fasting. The effects of fasting were comparable in men and women. To our knowledge, this is the first study that evaluated the effects of fasting on systemic lipid/metabolite levels in GB patients. Our results may hold promise for integrating fasting interventions as a component of a potential metabolic tumor therapy.

## Graphical abstract

Presurgery 72‐h fasting in GB patients leads to adaptations of plasma lipids and polar metabolites. Fasting reduces lysophosphatidylcholines and increases free fatty acids, shifts triglycerides toward long‐chain TGs and increases branched‐chain amino acids, alpha aminobutyric acid, and uric acid.

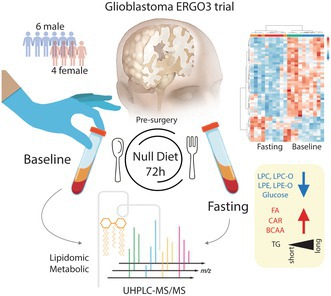

## Abbreviations

**Untargeted lipid classes**
CE: cholesterolester
Cer: ceramides
Chol: cholesterol
DG: diacylglycerol
FA: fatty acid
HexCer: hexosylceramides
LPC: lysophosphatidylcholine
LPC‐O: lysophosphatidylcholine ether
LPI: lysophosphatidylinositol
LPS: lysophosphatidylserine
PC: phosphatidylcholine
PC‐O: phosphatidylcholine‐ether
PE: phosphatidylethanolamine
PE‐O: phosphatidylethanolamine‐ether
PG: phosphatidylglycerol
PI: phosphatidylinositol
PS: phosphatidylserine
SM: sphingomyelin
TG: triacylglycerol

**LC‐ESI‐MS/MS**
IS: internal standard
LLOQ: lower limit of quantification
*m*/*z*
: mass transition
PQN: pooled quality control normalization
QC: quality control
UHPLC: ultrahigh‐performance liquid chromatography
ULOQ: upper limit of quantification

**Statistics**
ANOVA: analysis of variance
CanDisc: canonical discriminant score
CI: confidence interval
DA: discriminant analysis
MANOVA: multivariate analysis of variance
PCA: principal component analysis
PLS: partial least square analysis

**Other abbreviations**
BCAA: branched‐chain amino acids
BMI: body mass index
PUFA: polyunsaturated fatty acids

## 1 Introduction

The first mentions of health benefits of fasting date back more than a thousand years. Reprogramming of energy metabolism has been recognized as an emerging hallmark of cancer, and consequently, fasting and dietary interventions have gained much attention in oncology [1]. Diffuse gliomas and especially glioblastomas (GB) are the most common primary malignant brain tumors in adults with a poor prognosis despite multimodal therapy [2]. Therefore, new therapeutic approaches are urgently needed. During fasting, metabolism is expected to shift toward ketosis with some reduction in blood glucose [3]. Brain tumor cells have long been assumed to meet their energy demand mainly from glucose; however, this has recently been challenged when fatty acids were identified as a readily consumable energy source in gliomas [4]. GB cells can utilize systemic lipid resources for energy production [5, 6] because they are able to take up lipid‐laden extracellular vesicles [7, 8, 9]. In addition to providing energy, lipids are important building blocks of many cellular components, including membranes, which allow cells to proliferate. To meet the high lipid demands, fatty acid *de novo* synthesis via fatty acid synthase (FASN) is upregulated in glioma cells [10, 11, 12], and FASN expression levels have been shown to increase with the grade of malignancy [13]. At the same time, lipotoxicity is prevented by upregulation of diacylglycerol‐acyltransferase that transfers excess free fatty acids into triglycerides and lipid droplets [14] that serve as intracellular stores for fatty acids and cholesterol under conditions of starvation [15]. Most organs or tissues acquire lipids from the diet or from hepatic lipogenesis via the bloodstream [16, 17], but the mammary gland and the brain do not, and therefore, GB heavily depend on FASN mediated *de novo* synthesis.

Preclinical models have investigated the therapeutic potential of pharmacological interventions targeting lipid metabolism in glioma. In glioblastoma‐bearing mice, radiotherapy was found to promote lipogenesis in the mouse glioma microenvironment, which was associated with radio‐resistance [10]. Consequently, pharmacological inhibition of FASN along with irradiation improved survival of tumor bearing mice. In another study, enhanced fatty acid oxidation was associated with enhanced GB growth that was impaired by targeting fatty acid oxidation [18]. Apart from direct effects on the nutrient landscape of the brain, fasting‐mediated changes in peripheral lipids and metabolites may also indirectly affect brain tumors via stimulation of immune cells [19, 20] and modulation of the microbiome [21]. Considering the high lipid demand of GB, dietary changes or pharmacological interventions targeting lipid metabolism are under evaluation as add‐on treatment to the current standard of care. Indeed, a recent study revealed that GB is susceptible to FASN inhibition [22], revealing a potential metabolic vulnerability and encouraging the search for novel therapeutic interventions [23, 24].

There is increasing evidence that fine‐tuning of nutrients may sensitize cancer cells including GB to conventional cancer therapies [25], because irrespective of their heterogeneity, a shared trait is the alteration of metabolism [1, 26]. Several case studies and small clinical trials suggested that restricting glucose availability via a ketogenic diet may augment the efficacy of GB standard therapy [27, 28, 29, 30]. Short rounds of complete fasting (with free access to non‐caloric fluids) may even be more effective and induce an energy deprivation state in tumor cells that renders them more vulnerable to chemotherapy and immune reactions [19, 31]. In support of this concept, consumption of a high‐fat diet resulted in fatty acid accumulation and hyperaggressive disease, enrichment of glioma stem cells, and shortened survival in syngeneic and patient‐derived GB mouse models [32]. However, it is unknown to what extent systemic depletion of specific lipid classes or species, or a raise of ketone bodies affect glioma cells and stem cells in the tumor environment and if this results in a therapeutic benefit. Additionally, the individual's response to fasting will most likely depend on sex, age, and prefasting body composition and body mass index (BMI), comorbidities and drug treatment such as steroids that are commonly administered in brain tumor patients.

Previous lipidomic and metabolomic studies aimed to define “biomarkers” in human plasma that predict anticancer treatment efficacy, metastasis, or relapse [33, 34, 35, 36, 37]. In particular, ceramides have been correlated with various types of cancer [34, 38, 39]. Recently, plasma lipidomic patterns and specific sphingomyelins were suggested as prognostic markers of breast, ovarian, and pancreatic cancer [35, 40, 41], and a lipidomic panel of 11 lipid species was used to ascertain a diagnosis of malignant glioma [42]. It is important to note that plasma lipidomic patterns depend on the interaction of “age × sex,” which might impact on the efficacy of fasting regimens [43, 44, 45, 46, 47, 48, 49, 50, 51].

Owing to the complexity of the plasma lipidome, circadian rhythms, the transient nature of lipid metabolites and interconversion as well as the individuality of lifestyle and behavior, it is unknown how individual glioma patients tolerate and respond to a short course of 72‐h preoperative (biopsy or resection) fasting period. Here, we report on the changes in plasma lipidomic and metabolomic profiles of 10 glioma patients who, as part of the ERGO3 trial (NCT04461938), completed a 72‐h fasting cycle prior to tumor surgery.

## 2 Methods

### 2.1 Patient recruitment

The patients participated in the ERGO3 trial, which was a single‐arm clinical trial designed to characterize metabolic changes in the glioma tumor tissue induced by transient fasting. The trial intervention consisted of one fasting period of 72 h prior to biopsy or resection with unlimited intake of calorie‐free fluids such as water, tea, or coffee without sugar. Artificial sweeteners were allowed. Prior to the 72‐h fasting period, patients adhered to their normal individual diet and were not advised to change diets. Between March 2020 and December 2022, a total of 30 patients were enrolled into the trial. Blood plasma samples were obtained before and after the 72‐h fasting period of 10 patients (*n* = 6 men, *n* = 4 women) for lipidomic and metabolomic analyses. Patients' demographic data are presented in Table 1.

**Table 1:** Demographic data. BMI, body mass index; IDH, Isocitrate dehydrogenase; MGMT, O^6^‐methylguanine‐DNA methyltransferase.

Pat ID	Sex	Age (years)	BMI (kg·m^−2^)	Diagnosis	MGMT promotor methylation	First‐line treatment	Survival (months)
17	Male	62	25.4	GB, IDH‐Wildtype	Methylated	Resection followed by radiochemotherapy	17 months (deceased)
18	Female	62	29.7	GB, IDH‐Wildtype		Radiochemotherapy	41 months (deceased)
19	Male	68	21.8	GB, IDH‐Wildtype	Methylated	Resection followed by radiotherapy	35 months (alive)
20	Male	59	27.5	GB, IDH‐Wildtype	Unmethylated	Resection followed by radiochemotherapy	22 months (deceased)
21	Male	77	24.8	GB, IDH‐Wildtype	Methylated	Resection followed by radiochemotherapy	26 months (deceased)
22	Male	64	25.5	GB, IDH‐Wildtype	Unmethylated	Loss of follow‐up	Loss of follow‐up
23	Male	61	29.7	Diffuse pediatric‐type high‐grade glioma	Unmethylated	Resection followed by radiochemotherapy	20 months (deceased)
24	Female	62	21.8	GB, IDH‐Wildtype	Unmethylated	Resection followed by radiotherapy	33 months (alive)
29	Female	72	30.9	GB, IDH‐Wildtype	Methylated	Resection followed by radiochemotherapy	27 months (alive)
30	Female	55	22.0	GB, IDH‐Wildtype	Unmethylated	Resection followed by radiochemotherapy	16 months (alive)

Inclusion criteria were a suspected new diagnosis of glioma or suspected relapse of a previously diagnosed glioma based on MRI, and an interdisciplinary tumor board recommendation for resection or biopsy. Exclusion criteria were insulin‐dependent diabetes, dexamethasone at doses of > 4 mg·day^−1^, severe acute infection or immunosuppression, malnutrition or underweight with body mass index (BMI) < 18, hyper‐ or hypothyroid hormone levels, pancreatic insufficiency, and dementia or other clinically relevant alterations of the mental status.

The ERGO3 study was approved by the Ethics Committee at the University Hospital Frankfurt (project no. 19‐453). All patients provided written informed consent for the participation in the trial as well as for the pseudonymized processing and publication of their data. The trial was registered at clinicaltrials.gov (NCT04461938) and conformed to the standards set by the Declaration of Helsinki.

### 2.2 Blood sample collection

Blood samples were collected in K3‐EDTA tubes (Microvette Sarstedt, Nümbrecht, Germany) at baseline in the morning after overnight fasting and at completion of the 72‐h fasting period. Samples were centrifuged in a tabletop centrifuge (Eppendorf, Hamburg, Germany) at 1500 ***g*** and 4 °C for 10 min within 20 min of sampling. Plasma aliquots were immediately frozen after centrifugation and kept temporarily at −20 °C and then at −80 °C until analysis.

### 2.3 Untargeted lipidomic analyses

The analysis of lipids and polar metabolites was conducted using an UHPLC‐HRMS screening method as detailed in [52]. In summary, 10 μL of plasma underwent liquid–liquid extraction (LLE) with 75 μL of internal standards in methanol, 250 μL of methyl tert‐butyl ether (MTBE), and 50 μL of 50 mm ammonium formate. After vortexing and centrifugation, the upper phase was collected. The lower phase was re‐extracted with 100 μL of a MTBE:methanol:water (10 : 3 : 2.5) mixture. For lipid analysis, the upper phases were dried, stored below −70 °C, and reconstituted in 100 μL methanol. For polar metabolites, the lower phase was dried and stored below −70 °C. Quality control (QC) samples were pooled from the unknown samples.

The instrumentation included a Vanquish Horizon UHPLC system and an Orbitrap Exploris 480 operating in positive and negative ionization modes. Lipid profiling used a Zorbax RRHD Eclipse Plus C8 column (2.1 × 50 mm, 1.8 μm) with a guard column of the same type (Agilent) and a gradient elution of solvent A (10 mm ammonium formate +0.1% formic acid in water) and solvent B (0.1% formic acid in acetonitrile:isopropanol, 2 : 3). Data acquisition was done using XCalibur v4.4 and TraceFinder v5.1 (Thermo Fisher Scientific). Full scan spectra were acquired with a resolution of 120 000 at a mass range of 180–1500 *m*/*z* and a resolution of 15 000 for data‐dependent MS/MS spectra (ddMS^2^). Lipidomics results were normalized using one internal standard per lipid class.

Analyzed metabolites and lipids were identified with a mass error of ±5 ppm, by the assessment of the isotope ratio and by matching acquired MS/MS spectra to library spectra.

### 2.4 Untargeted analyses of polar metabolites

For metabolomic analysis, lower phases were reconstituted in 100 μL of acetonitrile:water (1 : 1). Polar metabolites were separated on an SeQuant ZIC‐HILIC, 3.5 μm, 100 mm × 2.1 mm I.D. column coupled to a guard column with equal chemistry (both Merck, Darmstadt, Germany) and a KrudKatcher inline filter (Phenomenex, Aschaffenburg, Germany). Using 0.1% formic acid in water (solvent A) and 0.1% formic acid in acetonitrile (solvent B), binary gradient elution was performed with a run time of 25 min. Full scan spectra were acquired with a resolution of 120 000 at a mass range of 70–700 *m*/*z* and 59–590 *m*/*z* in positive and negative ionization modes, respectively. The ddMS^2^ spectra were acquired at a resolution of 15 000. Relative quantification was performed based on peak areas in extracted ion chromatograms, normalized by median‐based probabilistic quotient normalization (PQN).

### 2.5 Statistics

Peak areas of chromatograms (AUCs) of untargeted lipidomic analyses were normalized to the AUC of internal standards (AUC/IS). Hence, the raw data are the AUC ratio versus IS. For the polar metabolites, the normalized chromatographic peak area was used. These data are presented as scatter plots with mean ± standard deviation (SD) or box‐scatter plots, where the box is the interquartile range and the whiskers show minimum to maximum, or the 95% confidence interval (CI), specified in the figure legend. Data were square‐root (SQR)‐transformed to adjust a skewed distribution. For heat maps, data were scaled to have a common mean and standard deviation of 1 (*z*‐scores), referred to as autoscaling in MetaboAnalyst ((x−μ)/SD, where μ is the mean). Data were analyzed with spss 29 (IBM SPSS Statistics) (RRID:SCR_002865) (MANOVA, canonical discrimination analyses, linear correlation, and mixed linear models), origin pro 2024 (OriginLab Corporation, Ditributor Additive GmbH. Friedrichsdorf, Germany) (RRID:SCR_014212), graphpad prism 9 (GraphPad Software, Boston, MA, USA) (RRID:SCR_002798), and metaboanalyst (https://www.metaboanalyst.ca/) (PLS‐DA) (RRID:SCR_016723).

To test the null‐hypothesis that metabolites before and after fasting were identical, paired t‐tests were used for lipid classes or MANOVA using nutrition state (before/after) and lipid or polar metabolite as within subject factors. In case of significant differences, pre/postfasting groups were compared for each feature (lipid or polar metabolite) using *post hoc* t‐tests according to Šidák or Fals Discovery Rate (FDR). Partial least square discrimination analysis (PLS‐DA) was used to reduce dimensionality and identify the features (analytes), which contributed most to the variance, and discriminated best between groups. The nutrition state (prefasting versus postfasting) were considered as paired data (within subject factor time point), and sex was introduced as covariate in some analyses. In addition, linear canonical discriminant analysis (DA) was used to assess the predictability of group membership based on DA scores. DA was performed without and with bootstrapping, the latter using a stratified random sampling approach considering sex and age, and 100 iterations. Further analyses included Random Forest to assess the predictability of group membership. For PLS‐DA and Random Forest, lipids (or polar metabolites) were classified according to their importance (VIP plots). Feature correlations (Pearson) that matched defined pattern were used to further dissect features responding to fasting and features showing sex differences. Volcano plots were used to assess fold differences of lipids or polar metabolites versus the negative logarithm (Log_10_) of the *t*‐test *P*‐value according to standard procedures. The alpha level was set at 0.05 for all comparisons.

## 3 Results

### 3.1 Lipidomic profiles of lipid classes before and after 72‐h fasting

**Fig. 1:**
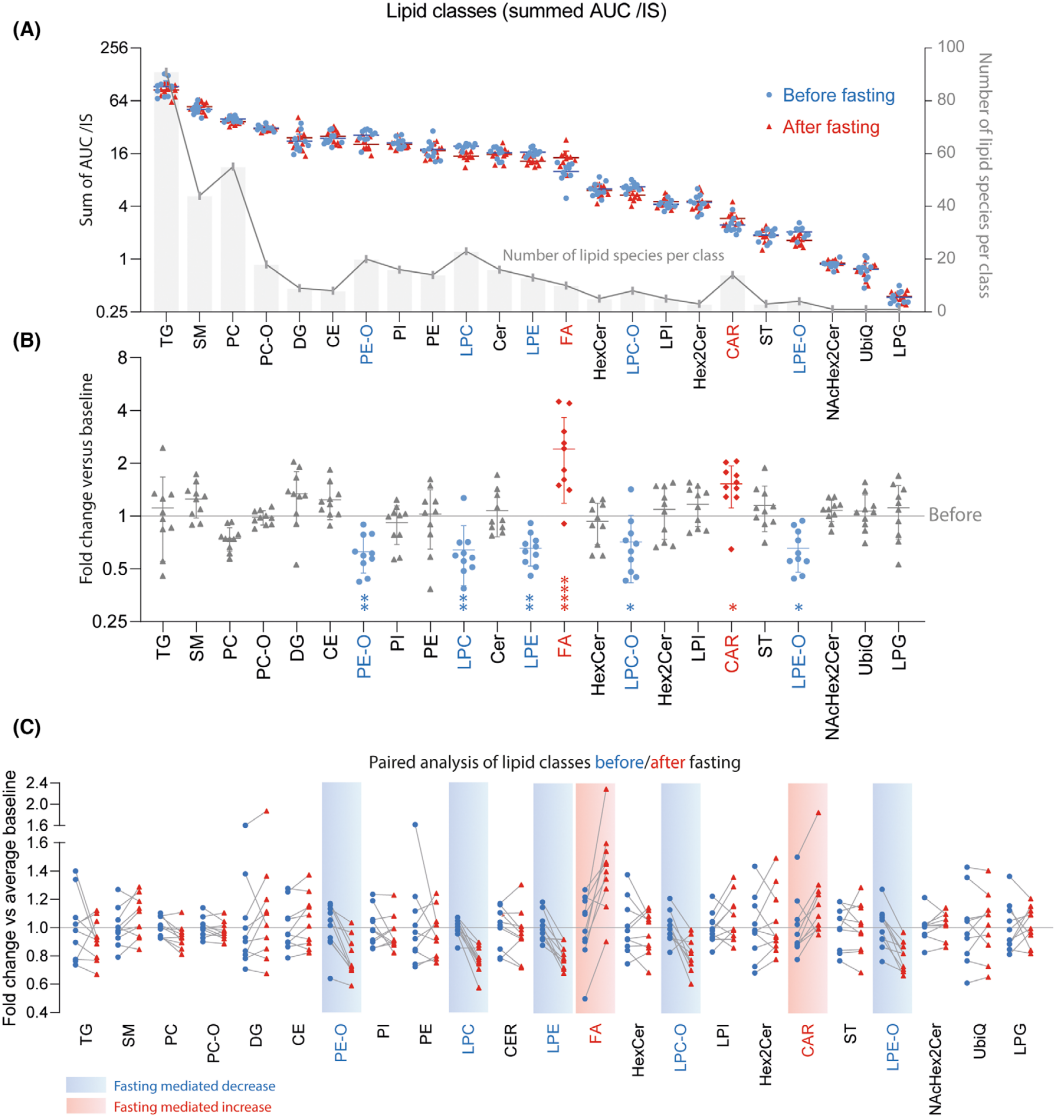
Effects of 72‐h fasting on plasma levels of lipid classes in GB patients subjected to a presurgery null‐diet. Plasma samples were obtained from *n* = 10 patients before and at completion of 72‐h fasting (referred to as after fasting) and subjected to lipidomic and metabolomic mass spectrometry analyses. (A) Areas under the MS peaks were normalized with the AUC of the respective internal standard (IS), expressed as AUC/IS. The AUC/IS values of lipid species were summed per lipid class. Lipid classes are sorted from left to right according to the numbers of species. The line is the mean, whiskers show the SD. Lipid classes reduced upon fasting are in blue letters, increased classes are in red. (B) Before/after fasting AUC/IS values were transformed to a ratio versus the individual's baseline to assess the extent of the fasting‐evoked fold change of the lipid classes. The baseline before fasting value is indicated with the line at Fold = 1. Lipid classes which were significantly reduced are shown in blue, significantly increased lipid classes are shown in red. The line is the mean, whiskers show the SD. Data were compared by two‐way ANOVA using “time‐point” X “lipid class”. *P* < 0.05. Sex‐specific plots are shown in Fig. S1. (C) Paired analysis of before and after fasting data. Blue shadowed lipid classes were significantly reduced, red shadowed significantly increased. CAR, carnitines; CE, cholesterol ester; CER, ceramides; DG, diglycerides; FA, fatty acids; HexCer, hexosylceramides; LPC, lysophosphatidylcholines; LPE, lysophosphatidylethanolamines; LPG, lysophosphatidylglycerols; LPI, lysophosphatidylinositols; –O, ether bound; PC, phosphatidylcholines; PD, phosphatidylglycerols; PE, phosphatidylethanolamines; PI, phosphatidylinositols; SM, sphingomyelins; ST, sterols; TG, triglycerides; UbiQ, ubiquitin.

For analysis of bulk lipidomic effects, AUC/IS ratios of lipid species were summed to obtain insight into changes in lipid classes (Fig. 1A). Because the abundance of lipids differs by several orders of magnitude between groups, differences are better noticeable after transformation into ratios “fasting” versus “baseline,” which was set to 1 (Fig. 1B, Fig. S1). The comparison shows that lysophosphatidylcholines (LPC), (lyso)‐phosphatidylethanolamines (PE and LPE), and their ether bound derivatives (LPC‐O, LPE‐O) are reduced after fasting, whereas free fatty acids and carnitines are increased in line with an expected utilization of neutral lipid stores for energy production. Sex‐specific analyses (Fig. S1A men, S1B women) corresponding to Fig. 1B show that the relative changes in lipid classes are comparable in men and women. Further details of the individual's changes are shown as paired data analysis (Fig. 1C).

**Fig. 2:**
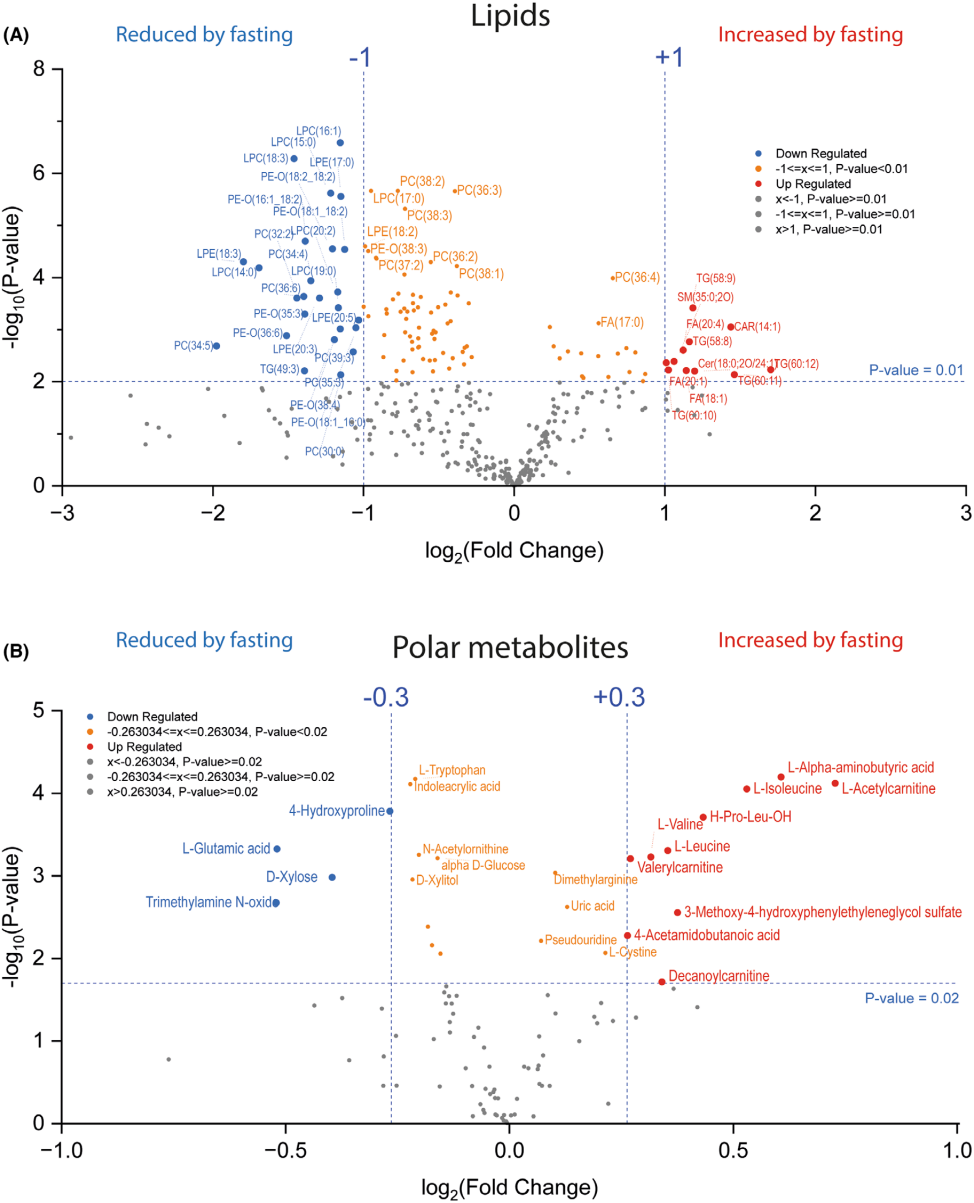
Volcano plots of lipids and polar metabolites in *n* = 10 GB patients before and at completion of 72‐h fasting. (A) Volcano plot comparing lipid species before and after fasting. The thresholds were set to two‐fold change (Log2‐fold change −1; +1) and *t*‐test *P* = 0.01. Lipids reduced after fasting are in blue (left), increased after fasting in red (right). Lipids with significant changes upon fasting (according to unpaired, two‐sided *t*‐test) but below the fold change threshold are in orange. Abbreviations as in Fig. 1. Numbers in () show C‐chain lengths and unsaturated bounds. (B) Volcano plot comparing polar metabolites before and after fasting. The thresholds were set to 25% change (Log_2_‐fold change −0.3; +0.3) and t‐test *P* = 0.02. Color definitions as in A.

To assess changes in specific lipids and of polar metabolites, Log_2_‐fold changes were plotted versus the negative logarithm of the *P*‐value as Volcano plots (Fig. 2). Multiple lipids are reduced while only few are increased (Fig. 2A). Six of the top 10 downregulated lipids are LPCs of different chain lengths and saturation. Some free fatty acids and very long‐chain triglycerides are upregulated compatible with an exploitation of lipid stores. Regarding polar metabolites, we found an increase in branched‐chain amino acids (Valine, Leucine, Isoleucine) and of alpha aminobutyric and aminobutanoic acid (Fig. 2B), which are expected to increase during fasting [53]. Similar to the increase in long‐chain carnitines seen with lipidomic analyses, metabolomic data show an increase in short‐chain carnitines, overall suggesting preferential ATP generation from lipids via beta‐oxidation.

### 3.2 Discrimination of prefasting versus postfasting conditions

**Fig. 3:**
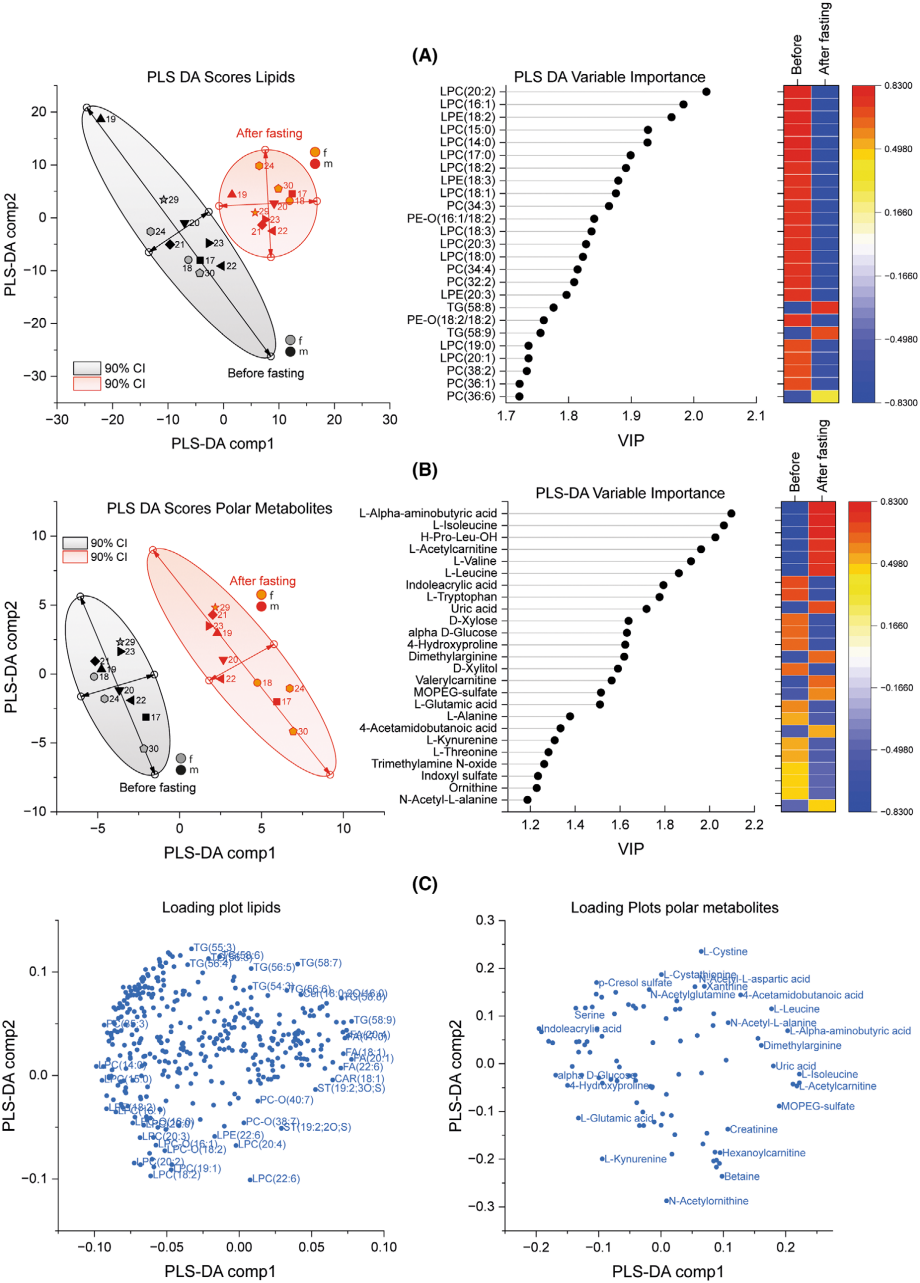
Partial Least Square Discrimination analysis of lipids and polar metabolites before/after fasting. (A) Score plots of PLS‐DA for lipid species for Component‐1 versus Component‐2 and the variable importance plot (VIP). (B) Score plots of PLS‐DA for polar metabolites for Component‐1 versus Component‐2 and the variable importance plot (VIP). The scatters show individual patients. The number is the subject‐ID, and sex is shown in different symbol colors (f = female, m = male). Shaded ellipses show the 90% confidence intervals (CI). (C) PLS‐DA loading plots for lipid species and for polar metabolites.

Partial least square discrimination analysis was used to assess whether the prefasting and postfasting conditions were discriminable and to rank the importance of the features (Fig. 3). Score plots of the first two dimensions (Component‐1 and ‐2) show separate clusters for the pre‐ and postfasting conditions with nonoverlapping 90% confidence intervals (Fig. 3A lipids, 3B polar metabolites). Variable importance plots (right) show that the leading lipid features are LPCs which, as reported above, are all decreased upon fasting. The leading polar metabolites are aminobutyric acid and branched‐chain amino acids, which are increased upon fasting. The results are in line with t‐test statistics of Volcano plots (Fig. 2).

### 3.3 Individual lipid and polar metabolomic profiles and fasting efficacy

**Fig. 4:**
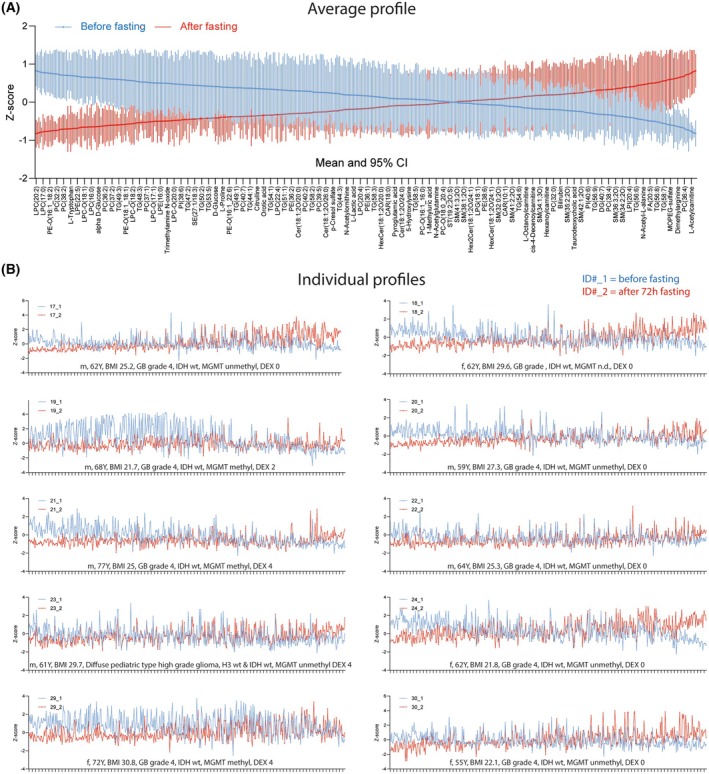
Average and individual profiles of lipids and polar metabolites in study participants sorted according to the extent of change versus baseline (before). (A) AUC/IS data were transformed to *Z*‐scores to have a common mean and variance of 1 (*Z*‐score = (*X*− *X¯*)/SD) and were sorted from left to right according to the extent of the fasting‐evoked change. Lipids/metabolites that were reduced by fasting are on the left side, increased by fasting right side. The lines show the average *Z*‐scores and the whiskers are the SD. (B) The sequence of the lipids and metabolites as in A (*x*‐axis as in A) was applied to each individual patient and the profiles show the lipids/metabolite *Z*‐score before (sample‐1, blue) and after fasting (sample‐2, red) for each patient. The patients' characteristics are displayed above the *X*‐axis. Demographic data are summarized in Table 1. BMI, body mass index; Dex, dexamethasone in mg per day; f, female; H3 wt, histone H3 wildtype; IDH wt, isocitrate dehydrogenase wildtype; m, male; Y, years; O6‐methylguanine‐DNA methyltransferase (MGMT) promoter methylation status methyl/unmethyl.

**Fig. 5:**
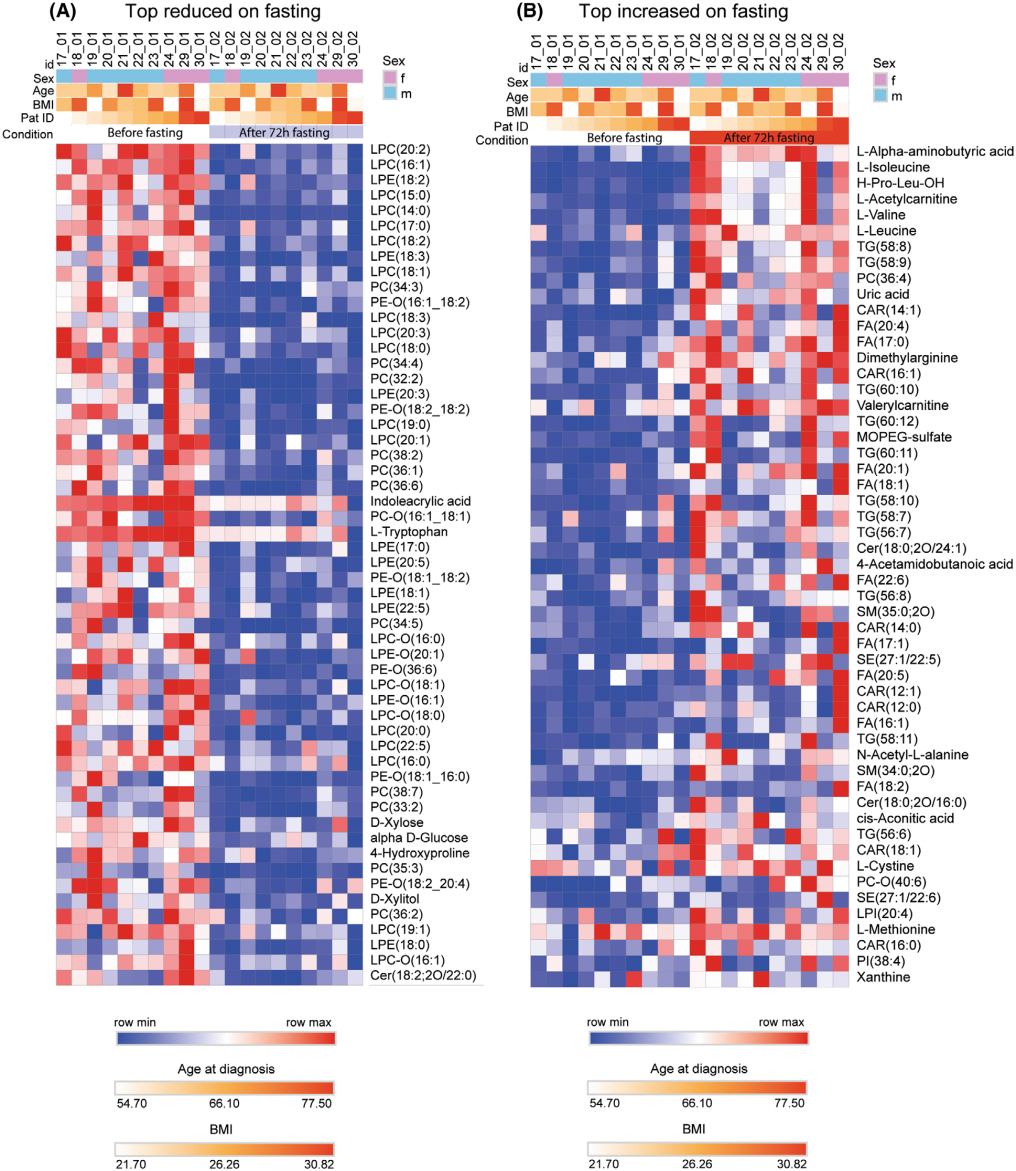
Heatmaps of top reduced (A) and top increased (B) lipids and polar metabolites upon fasting in consideration of the indicated demographic parameters. Lipids and polar metabolites were pooled and auto‐scaled. The color scales are shown at the bottom.

To assess individual profiles and the overall scheme, lipid and polar metabolite data were pooled and auto‐scaled to have a common mean and a variance of 1 (*Z*‐score). The features were sorted from most decreased to most increased upon fasting (average and SD in Fig. 4A), and the sequence was applied to each individual patient (Fig. 4B). The interindividual variability is higher at baseline than after fasting. Subjects 19 (male) and 29 (female) had high baseline triglycerides, which returned to normal upon fasting. Subjects 22 and 23 (both male) showed only minor effects of fasting. To further assess the impact of sex, age, and BMI, the top 50 downregulated and upregulated features were assessed in heatmaps (Fig. 5, Fig. S2). The heatmaps again identified LPC species as the top downregulated lipids, followed by some LPE species. For the upregulated features, the heatmap (right panel in Fig. 5) reveals higher interindividual variability as compared with the downregulated feature. The top candidates included aminobutyric acid, branched‐chain amino acids, long‐chain TGs and free fatty acids.

In Fig. 5, subjects are presented according to the sequence of recruitment, and sex is depicted as top meta data bar. To further assess the influence of “sex x fasting,” samples and features were clustered and presented as additional heatmaps in Fig. S2 (top 50 lipids) and Fig. S4 (top 25 polar metabolites). The analysis shows that fasting is the major determinant, and sex is only relevant for sphingomyelin species, which were however not affected by fasting. A pattern search (based on feature correlations, Fig. S3) again confirms that the major effect of fasting is a decrease in LPCs irrespective of sex (S3A), whereas sex differences are due to lower levels of sphingomyelin species in men than in women independent of fasting (Fig. S3B,C). Higher levels of plasma sphingomyelins in women than men agree with the expected age‐dependent increase in plasma sphingomyelins in women > 50 years of age [44, 46, 47, 51]. Overall, differences at baseline tend to decrease by fasting.

### 3.4 Candidate lipid species and candidate polar metabolites

**Fig. 6:**
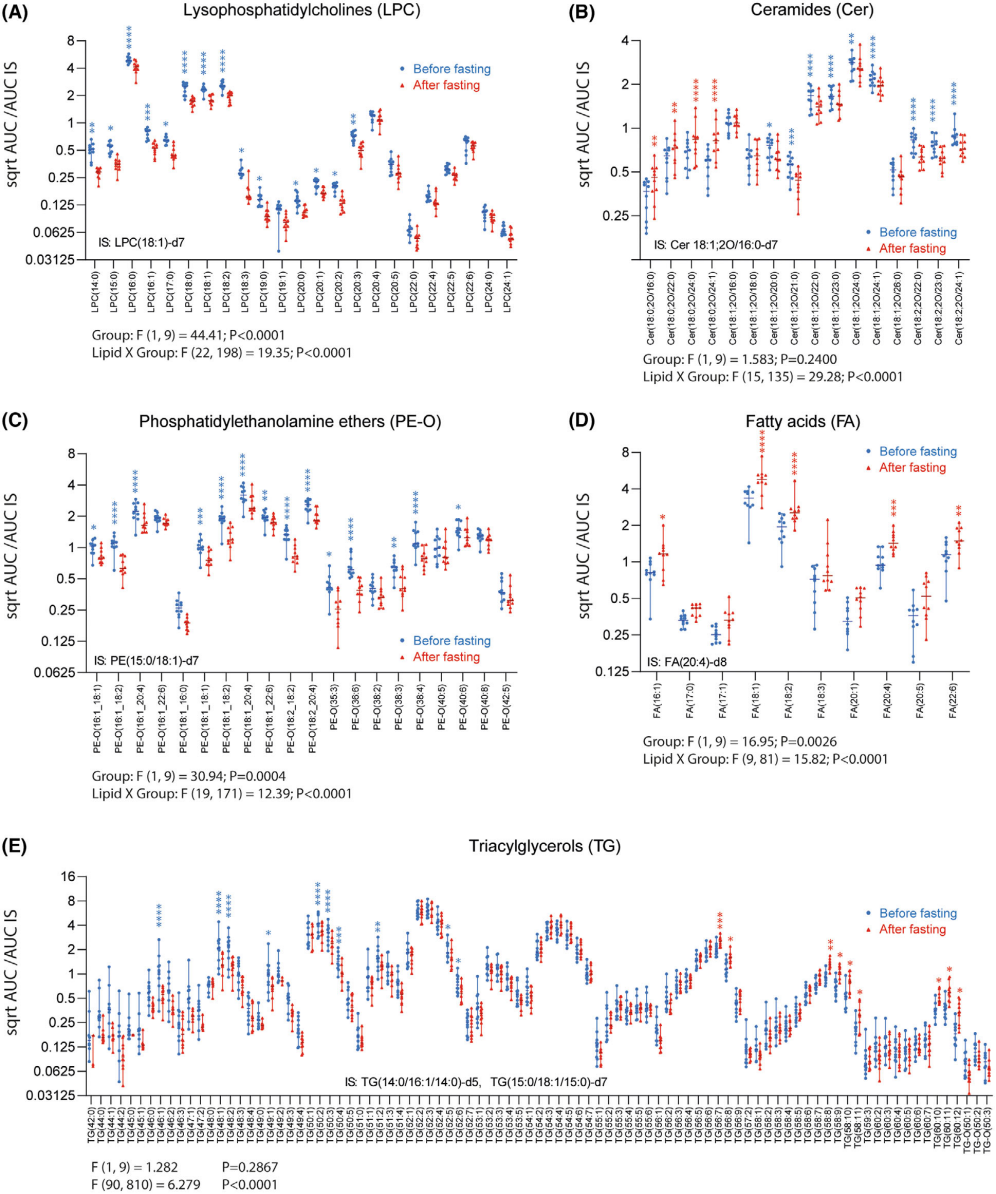
Scatter plots of lipid species of regulated lipid classes in *n* = 10 GB patients before and after fasting. The data show the mean ± SD of square root (sqrt) transformed AUC ratios of the analyte and the internal standard (AUC/IS, IS named in left corner in each panel). Sqrt transformation was used to straighten a skewed normal distribution. The scatters show individual patients. The asterisks indicate significant differences between fasting and baseline (ANOVA, *post hoc* Sidak, *P < 0.05, **P < 0.01, ***P < 0.001, ****P < 0.0001). (A) Lysophosphatidylcholines. (B) Ceramides. From left to right Cer d18:0 dehydro‐ceramides, Cer d18:1 ceramides, Cer d18:2 sphingadiene ceramides. (C) Phosphatidylethanolamine ethers. (D) Fatty acids. (E) Triacylglycerides (triglycerides).

**Fig. 7:**
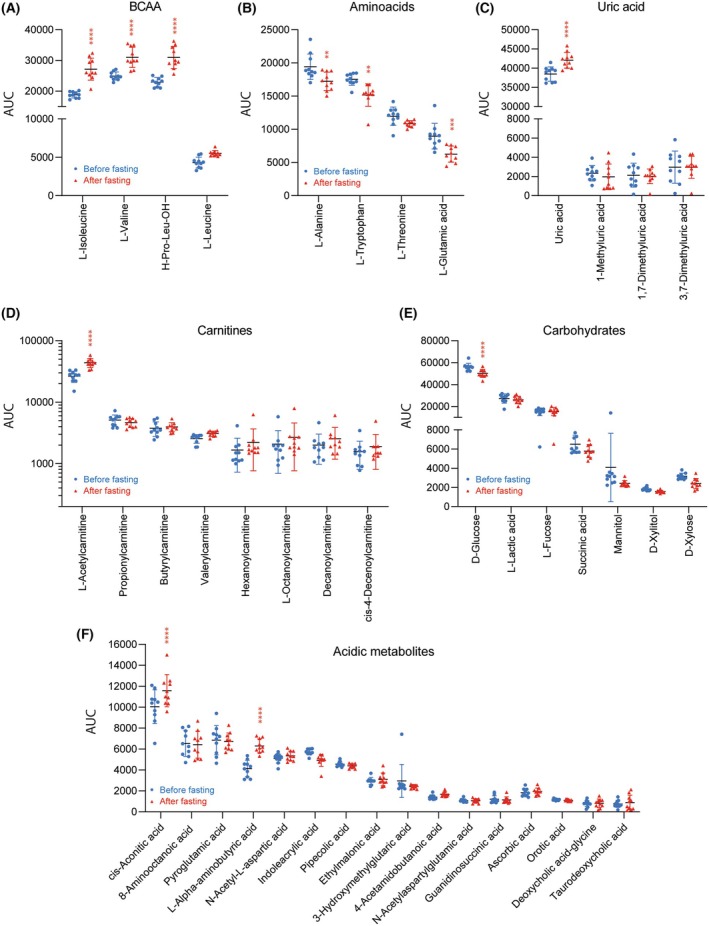
Scatter plots of candidate polar metabolites in *n* = 10 GB patients before and after fasting. The data show the mean ± SD of square root (sqrt) transformed AUCs. Sqrt transformation was used to straighten a skewed normal distribution. The scatters show individual patients. The asterisks indicate significant differences between fasting and baseline (ANOVA, *post hoc* Sidak, **P < 0.01, ***P < 0.001, ****P < 0.0001). (A) Branched‐chain amino acids (BCAA). (B) Amino acids. (C) Uric acid and metabolites. (D) Carnitines. (E) Carbohydrates. (F) Acidic metabolites.

To assess statistically significant differences of individual lipid species or polar metabolites, candidate species of candidate classes were submitted to two‐way ANOVA and post hoc analysis and plotted as scatter plots (Figs 6 and 7). The decrease in LPCs upon fasting applies to all LPC species (Fig. 6A). For ceramides, dehydro‐ceramides (Cer d18:0) were increased, indicating an increase in *de novo* synthesis, whereas Cer d18:1 species were reduced (Fig. 6B). PE‐O species were mostly reduced (Fig. 6C), whereas free fatty acids were increased (Fig. 6D). Triglyceride (TG) patterns were shifted from a reduction in short TGs to an increase in very long TGs, but the overall pattern of TGs (C‐chain length and saturation) was maintained (Fig. 6E). The profile change suggests that short‐chain TGs are more easily reutilized upon “starvation,” whereas very long‐chain TGs are preserved. Among polar metabolites, branched‐chain amino acids were increased (Fig. 7A), whereas other amino acids were not affected or even reduced such as glutamic acid (Fig. 7B). Uric acid increased as a result of ketogenesis (Fig. 7C), and L‐acetylcarnitine was increased (Fig. 7D). In contrast, carbohydrates were mostly unaffected except for a moderate yet significant decrease in glucose (Fig. 7E). L‐alpha‐aminobutyric acid was strongly increased (Fig. 7F), which is an expected positive effect of fasting [54, 55].

## 4 Discussion

The present study provides a detailed insight into the systemic plasma lipidomic and metabolomic effects of a 72‐h fasting period in patients with diffuse high‐grade gliomas in a small cohort of six male and four female GB patients. The fasting‐evoked changes in plasma lipids and polar metabolites in our patients showed a mild reduction in glucose, an increase in BCAA (valine, leucine, isoleucine) and uric acid, as well as an increase in fatty acids and carnitines, but decrease in (lyso)phosphatidylcholines (LPC, PC). Despite the small sample size, our observations are consistent with the current literature indicating that nutrient deprivation by fasting causes a mobilization of liver glycogen stores, catabolism of proteins reflected by an increase in plasma levels of BCAA, and exploitation of neutral lipid stores (triglycerides and cholesterol ester) via lipolysis and lipophagy, and subsequent β‐oxidation of fatty acids once glycogen stores are depleted [56]. This metabolic switch leads to an increase in ketone bodies (acetone, acetoacetate, and 3‐beta‐hydroxybutyrate), metabolic acidosis, reduction in urine pH, and increase in uric acid [57] owing to reduced renal tubular secretion [58]. The metabolic response is orchestrated by agouti‐related protein (AgRP)‐positive neurons in the hypothalamus [59]. After 3 days of fasting, about 25% of the brain's energy requirements are supposed to be met by ketone bodies [60], but unlike in healthy brain regions, ketone bodies accumulate in brain tumors [61] potentially because of abnormally high alternative neutral lipid stores [14, 62, 63].

The preliminary analysis of MR spectroscopy data of the ERGO3 study has shown that β‐hydroxybutyrate and acetoacetate were detectable in gliomas of all patients who completed the 72‐h fasting cycle [64], thus corroborating the metabolic effects reported here. Likewise, other studies based on MR spectroscopy have detected ketone bodies in tumors in GB patients under a ketogenic diet [61, 65] but also showed that energy homeostasis was maintained [66], hence suggesting that gliomas utilize other energy sources. An important predictor of GB chemotherapy efficacy is the methylation status of the MGMT gene promoter. The MGMT gene encodes for a DNA repair enzyme that can mitigate tumor cell apoptosis [67]. Quite interestingly, MGMT promoter methylation status may also impact the tumor's ability to maneuver lipid metabolism. Unmethylated MGMT status has been associated with increased lipid biogenesis, lipid accumulation, and lipophagy [63, 68]. MGMT unmethylated tumors showed stronger accumulation of long‐chain unsaturated fatty acids [63, 68]. In the periphery and healthy brain, intermittent fasting induces the cellular exploitation of neutral lipid stores via lipophagy, mostly triglycerides and cholesterol esters, but the MGMT unmethylated subclass of GB tumors appears to be impaired in this metabolic adaptation. While our cohort is representative in that the majority of GB tumors lacked MGMT methylation, the small number of patients did not allow for stratification of metabolic responses to fasting in relation to MGMT promotor status.

Increased plasma/serum levels of BCAAs levels are associated with insulin resistance [69], which is mechanistically still not completely understood. BCAAs are elevated in diabetes and obesity [53, 70], and high‐protein diets enriched with BCAA cause acute insulin resistance [71]. Conversely, pharmacologic activation of branched‐chain α‐ketoacid dehydrogenase, the rate‐limiting enzyme of BCAA oxidation, lowers plasma BCAAs, and improves insulin sensitivity [72]. It has not been studied whether fasting‐evoked increased levels of BCAA are readily available for GB cells, but it is known that the expression of large neutral amino acid transporter 1 (LAT1, SLC7A5) that imports BCAA through the blood brain barrier is increased in GB [73, 74]. In addition, branched‐chain amino acid transaminase 1 (BCAT1) is upregulated in GB resulting in a high rate of BCAA conversion to branched‐chain α‐ketoacids [75], which promote immunosuppression [76, 77] and tumor growth [78, 79, 80]. Hence, although not proven, a fasting‐evoked BCAA release from muscle and liver is most likely not a therapeutic advantage.

We show that LPCs and LPEs and their ether bound derivatives are decreased after fasting while free fatty acids and carnitines are increased (Figs 6 and 7). The drop of LPCs is the most robust change and occurred in all patients. This is in line with data of a 36‐h water‐only fasting study in healthy young volunteers (f = 10, m = 10, age 27, BMI 24) [81]. Unfortunately, this paper only reported fold changes and *P*‐values of the metabolites. From the summary data, it seemed that the reduction in short‐chain TGs was stronger in the young cohort than in our patients, but was also associated with an increase in very long‐chain, polyunsaturated TGs (≥ 58 : 7). Hence, TG profiles show a shift from short‐chain to long‐chain TGs without an overall change of TG abundance suggesting that short‐chain TGs are more easily mobilized upon nutrient deprivation. Under physiologic conditions, TG levels in the brain are low unless the blood brain barrier is seriously disrupted (e.g., after traumatic brain injury), which then leads to a massive influx of plasma TGs that are taken up and stored in perilesional microglia [82]. Gliomas *per se*, surgical resection, and irradiation may also disrupt the BBB to an extent that allows influx of plasma TGs. Indeed, it was shown that GB irradiation in a mouse xenograft model induced a lipid‐enriched, growth‐permissive tumor microenvironment in association with radio‐resistance [10]. Lipid staining techniques presently do not allow to distinguish between TG species of different C‐chain length and saturation, and it is not known whether GBs favor short over long‐chain TGs, and whether a fasting‐induced shift toward long‐chain TGs in plasma may provide a therapeutic benefit.

A recent real‐time intraoperative ambient ionization mass spectrometry imaging (SpiderMass) study in GB patients identified some diglyceride species mainly in necrotic regions but not triglycerides [83]. Indeed, the absence of TG/DG was proposed as marker for healthy brain tissue [83]. Phospatidylserines (PS) were the most dominant lipid class in tumor regions, and high PS in combination with low phosphatidylcholines (PC) were associated with poor prognosis [83]. PS are enriched in membranes but low in plasma and, hence, PS species are not likely to be amenable to dietary interventions. However, it has to be considered that lipids may also be imported from the periphery via extracellular vesicles [84], which provide a source of PS and cholesterol, both needed to meet the high demand for membrane synthesis [85]. It may be possible to modify the lipid composition of plasma EV via fasting.

The catabolism of PCs and LPCs via patatin‐like phospholipase domain‐containing proteins (PNPLAs) provides an endogenous choline supply to replenish the methionine cycle with methyl groups [86]. The metabolic link between PC/LPC and C1 supply has been mainly studied in the liver [86]. Specifically, PNPLA7 acts as ER‐anchored PC/LPC hydrolase [87] that is required for interaction and utilization of lipid droplets, and elevated expression has been associated with gastrointestinal, liver and breast cancer [88, 89], but so far not in GB or other diffuse gliomas. LPCs need to be actively imported into the brain via a sodium‐dependent transporter called Mfsd2a (Major facilitator superfamily, MFS) [90], which is a crucial component of the blood brain barrier [91, 92]. Zwitterionic LPC headgroups bind long‐chain unsaturated fatty acids (PUFA), including docosahexaenoic acid and alpha linolenic acid, and carry them via Mfsd2a through the blood brain barrier and deliver PUFAs to the brain [93, 94].

Therefore, peripheral LPCs are essential as carriers for PUFAs into the brain and likely into the tumor. However, it is unclear whether GB cells including stem cells essentially need PUFAs like the normal brain [95, 96, 97]. In brain injury, high plasma levels of LPCs have been associated with survival and recovery in mice and in humans [98, 99]. One may hypothesize that the opposite, that is, low LPC might lead to tumor PUFA deprivation possibly augmenting the responsiveness toward anti‐GB therapy. Publicly available CGGA and TCGA GBM‐LGG data (http://gliovis.bioinfo.cnio.es/) show that low expression of Mfsd2a tends to be associated with longer survival. It is of note that Mfsd2a controls transcytosis independent of its lipid carrier‐like functions, and thereby blocks brain uptake of temozolomide [100, 101]. This effect may contribute to survival benefits of tumors with low Mfsd2a expression. Metabolically, high LPC has been associated with obesity [102], inflammation [103, 104] and cardiovascular disease [105] suggesting that fasting‐mediated lowering of plasma LPC likely provides an additional health benefit regardless of its potential effects on tumor growth or resistance.

It is of note that plasma LPC levels depend on a hormone‐sensitive “sex x age” interaction [45, 47, 48, 49]. Women in the reproductive age have higher plasma LPC levels than men, because LPCs are needed during all stages of female reproduction [106, 107]. Sex differences in plasma LPCs are fading upon aging and hence, no longer evident in our small cohort of GB patients who were > 55 years of age. Importantly, the fasting‐evoked lowering of plasma LPCs was comparable in men and women at this age. The interaction of “sex × age” is also a crucial determinant of plasma sphingomyelins, which steadily increase in women > 50 years of age [43, 44, 46]. Despite the small number of patients in our study, comparison of female versus male patients show indeed higher levels of the majority of sphingomyelin species in female patients (Figs S2 and S3), which were however not affected by fasting. Hence, SMs were able to differentiate between women and men but not between baseline versus fasting. The low number of participants in our study does not allow for statistical analysis of further covariates (e.g., BMI, age). Overall, it appears that interindividual variability in plasma lipid species that is present at baseline decreases with fasting (as indicated by smaller 95%‐CI) suggesting a synchronizing or equalizing effect. Studies in larger cohorts are needed for confirmation of the observed patterns.

## 5 Conclusions

In summary, this is, to our knowledge, the first profound lipidomic and metabolomic plasma analysis investigating metabolic effects of fasting in glioma patients. Our data reveal LPCs as the most striking regulated lipid subclass upon nutrient deprivation. Considering that peripheral LPCs are omega‐3 lipid carriers and precursors of lysophosphatidic acids, both key players in angiogenesis, immunosurveillance and cell mobility [108, 109], our results suggest that fasting‐mediated LPC lowering may be therapeutically relevant. Additional studies are necessary to investigate the potential of dietary interventions as a component of a metabolic glioma therapy.

## Conflict of interest

JPS reports honoraria for lectures or advisory board participation or consulting or travel grants from Abbvie, Roche, Boehringer, Bristol‐Myers Squibb, Medac, Mundipharma UCB and Servier. MWR reports a research grant from UCB as well as honoraria for advisory board participation from Alexion and Servier. All other authors declare no conflicts of interest.

## Author contributions

ID, KJW, and MWR recruited patients and collected samples. LH performed and supervised lipidomic and metabolomic analyses. MWR and JS conceived and designed the ERGO3 trial and obtained Ethics Approval and trial registration. GG acquired funding and resources. DK and IT acquired funding and ethics approval. IT compiled, analyzed, and interpreted data, wrote the paper, made the figures. All authors contributed to writing or editing parts of the manuscript and agree to the final version of the manuscript.

## Peer review

The peer review history for this article is available at https://www.webofscience.com/api/gateway/wos/peer‐review/10.1002/1878‐0261.70003.

## Supporting information


**Fig. S1.** Effects of 72‐h fasting on plasma levels of lipid classes in male and female GB patients.
**Fig. S2.** Heatmap of top 50 regulated lipids after 72 h fasting (after fasting) as compared with baseline (before fasting).
**Fig. S3.** Variable importance plots showing the top candidates accounting for effects of fasting and effects of sex.
**Fig. S4.** Heatmap of top 25 regulated polar metabolites after 72‐h fasting (after fasting) as compared with baseline (before fasting).

## Data Availability

All data that were analyzed for the study are presented within the manuscript or supplement. Raw source and processed data of UHPLC high‐resolution mass spectrometry analyses of lipids and polar metabolites and meta data are deposited at Goethe University Data repository GUDe with the DOI: https://doi.org/10.25716/gude.0r7b‐6vh$.
