# Supplementary material for: Plasma lipidomic and metabolomic profiles in high‐grade glioma patients before and after 72‐h presurgery water‐only fasting
Source: Mol Oncol. 2025 Feb 24;19(8):2249–69. doi: 10.1002/1878-0261.70003 (PMC12330934; doi:10.1002/1878-0261.70003)
Supplement: Supplementary file 1 — Fig. S1. Effects of 72‐h fasting on plasma levels of lipid classes in male and female GB patients. Fig. S2. Heatmap of top 50 regulated lipids after 72 h fasting (after fasting) as compared with baseline (before fasting). Fig. S3. Variable importance plots showing the top candidates accounting for effects of fasting and effects of sex. Fig. S4. Heatmap of top 25 regulated polar metabolites after 72‐h fasting (after fasting) as compared with baseline (before fasting). [file MOL2-19-2249-s001.pdf]

Supplementary Figures to:

Plasma lipidomic and metabolomic profiles in high grade glioma patients before and after 72 hours pre-surgery water-only fasting

Iris Divé[1-4], Lisa Hahnefeld[5,6], Katharina J. Wenger[2-4,7,8], Donat Kögel[3,9], Joachim Steinbach[1-4,8], Gerd Geisslinger[5,6], Michael W. Ronellenfitsch[1-4,8], Irmgard Tegeder[5]

1. Dr. Senckenberg Institute of Neurooncology, Goethe University Frankfurt, University Hospital, 60528 Frankfurt am Main, Germany
2. Center for Personalized Translational Epilepsy Research (CePTER), Goethe-University Frankfurt, University Hospital, Frankfurt am Main, Germany
3. University University Cancer Center Frankfurt (UCT), Goethe University Frankfurt, University Hospital, Frankfurt am Main, Germany
4. German Cancer Consortium (DKTK), Partner Site Frankfurt/Mainz, a partnership between DKFZ and University Hospital Frankfurt, Frankfurt am Main, Germany.
5. Institute for Clinical Pharmacology, Faculty of Medicine, Goethe University Frankfurt, 60590 Frankfurt am Main, Germany.
6. Fraunhofer Institute for Translational Medicine and Pharmacology ITMP and Fraunhofer Cluster of Excellence for Immune Mediated Diseases CIMD, 60596 Frankfurt am Main, Germany
7. Institute of Neuroradiology, Goethe-University Frankfurt, University Hospital, Frankfurt am Main, Germany
8. Frankfurt Cancer Institute (FCI), Goethe University Frankfurt, University Hospital, Frankfurt am Main, Germany
9. Department of Neurosurgery, Experimental Neurosurgery, Goethe-University Frankfurt, Neuroscience Center, 60528 Frankfurt am Main, Germany

Suppl. Figure 1

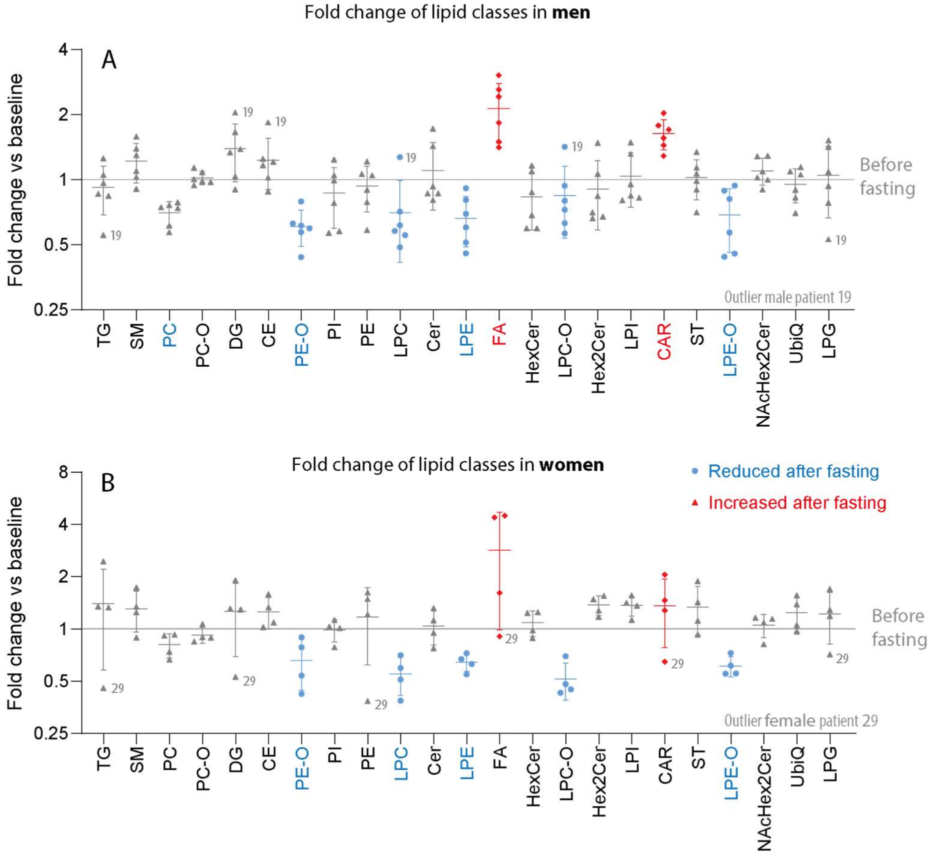

Figure S1

Effects of 72 h fasting on plasma levels of lipid classes in GB patients subjected to a pre-surgery null-diet. Plasma samples were obtained from n = 10 patients (6 male, 4 female) before and at completion of 72 h fasting (referred to as after fasting) and subjected to lipidomic and metabolomic mass spectrometry analyses. To reveal fasting-induced changes versus baseline before/after fasting, AUC/IS values were transformed to a ratio versus the individual's baseline to assess the extent of the fasting-evoked fold change of the lipid classes. The baseline level is indicated with the line at Fold=1. Data were compared by 2-way ANOVA using "time-point" X "lipid class" as shown in Figure 1 (main body) using all patients (n = 10). $P < 0.05$. Lipid classes which were significantly reduced are shown in blue, significantly increased lipid classes are shown in red. To assess sex-dependent differences in fasting effects the plots are presented for men (A) and women (B).

A, B show sex-specific fasting induced changes of lipid classes. The changes induced by fasting are comparable in men and women. Outlier spots depicting a male patient #19 and a female patient #29 are labeled. #19 had the lowest BMI among men (BMI 22), #29 had the highest BMI among women (BMI 31).

Abbreviations: CAR, carnitines; CER, ceramides; CE, cholesterol ester; DG, diglycerides; FA, fatty acids; HexCer, hexosylceramides; LPC, lysophosphatidylcholines; LPE, lysophosphatidylethanolamines; LPG, lysophosphatidylglycerols; LPI, lysophosphatidylinositols; PC, phosphatidylcholines; PE, phosphatidylethanolamines; PD, phosphatidylglycerols; PI, phosphatidylinositols; SM, sphingomyelins; ST, sterols; TG, triglycerides; UbiQ, ubiquitin; –O ether bound.

Suppl. Figure 2

Top 50 regulated lipids "fasting x sex"

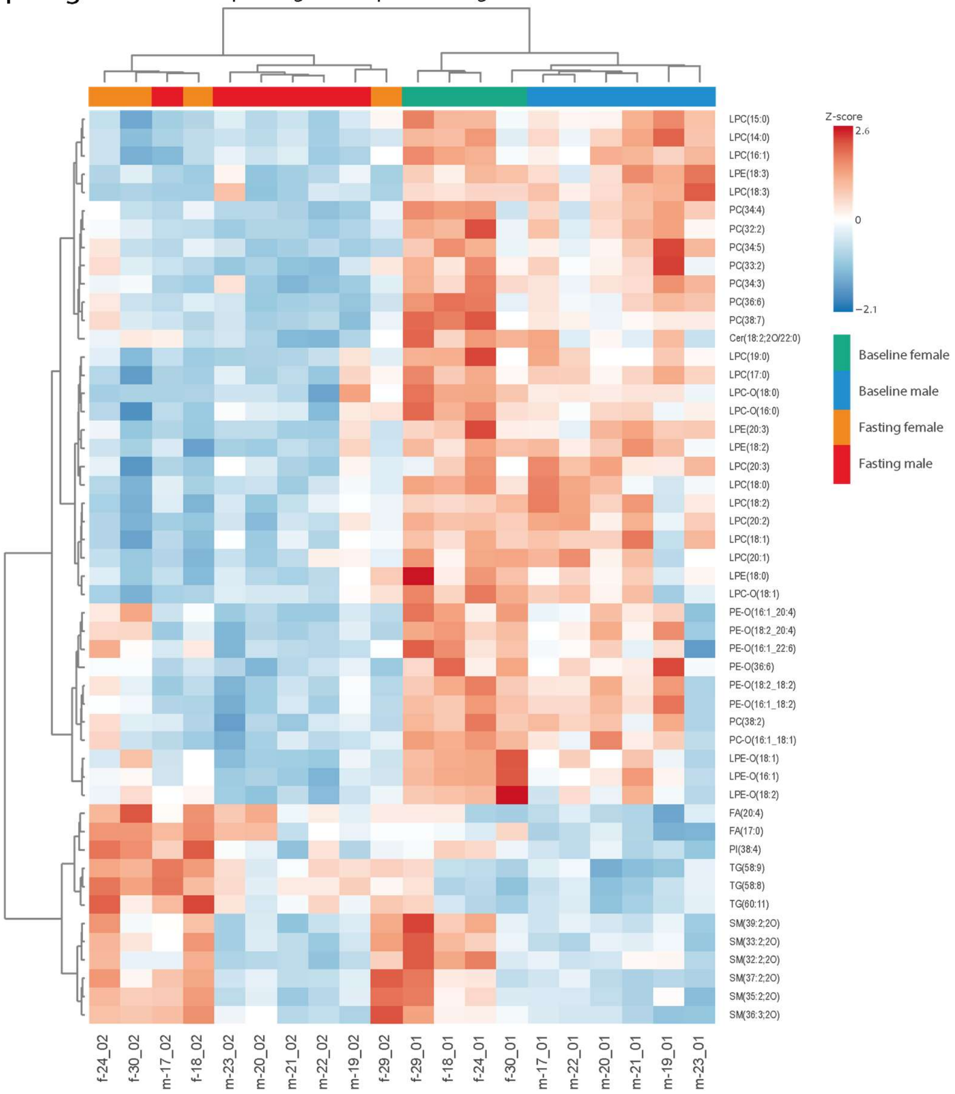

Figure S2

Heatmap of top 50 regulated lipids after 72h fasting (after fasting) as compared with baseline (before fasting). Rows (samples) and columns (lipid species) were clustered according to Euclidean distance metrics using the Ward method. The colors show auto-scaled data of the sqrt AUC/IS for each lipid species. Autoscaling refers to Z-transformation to a common mean and variance of 1. Columns cluster in Fasting and Baseline, mostly based on changes of LPC and PC species. At baseline, women and men are differentiated based on sphingomyelins, which are higher in women than men.

Abbreviations: f, female; m, male; AUC, area under the mass spectrometry curve, IS refers to the AUC of the internal standard, sqrt = square root. Abbreviations of lipids as in S1.

Suppl. Figure 3

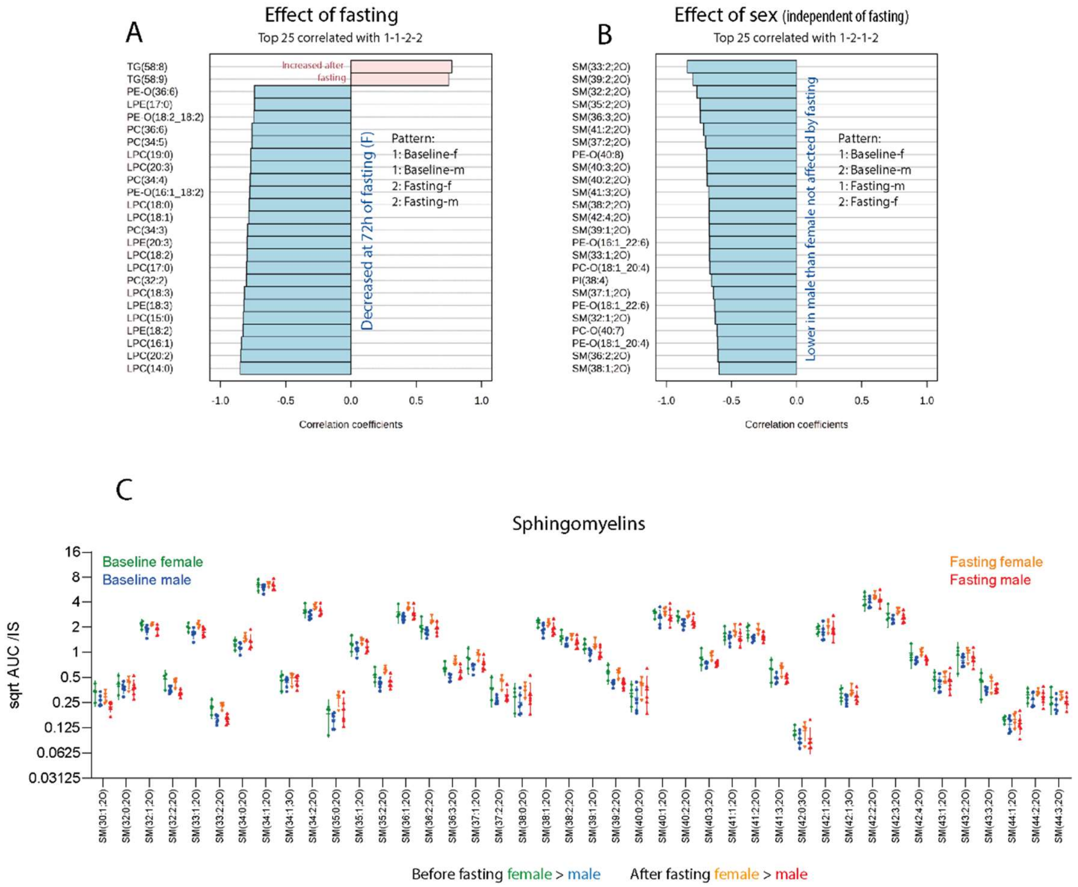

Figure S3

Variable importance plots showing the top candidates for effect of fasting and effect of sex. The selection of variables relies on ANOVA statistics using four groups, i.e. baseline-female (B-f), baseline male (B-m), fasting-female (F-f), fasting-male (F-m) and a pattern search

A: Top up and down regulated lipids fitting to the pattern 1-1-2-2 that describes differences between baseline (B-f, B-m) and fasting (F-f, F-m).

B: Top lipid candidates which fit to the pattern 1-2-1-2 searching for differences between women and men irrespective of fasting. The plots shows that male patients had lower sphingomyelin plasma levels than female patients which is the expected sphingomyelin (SM) sex-pattern at this age (>55-70+ years).

C: To further assess differences in sphingomyelins, SM species were plotted as scatter plots before and after fasting, and men/women are depicted in different colors. For most SM species, women have higher SM levels than men, before and after fasting. Fasting has no influence on the sex-dependent pattern.

Suppl. Figure 4

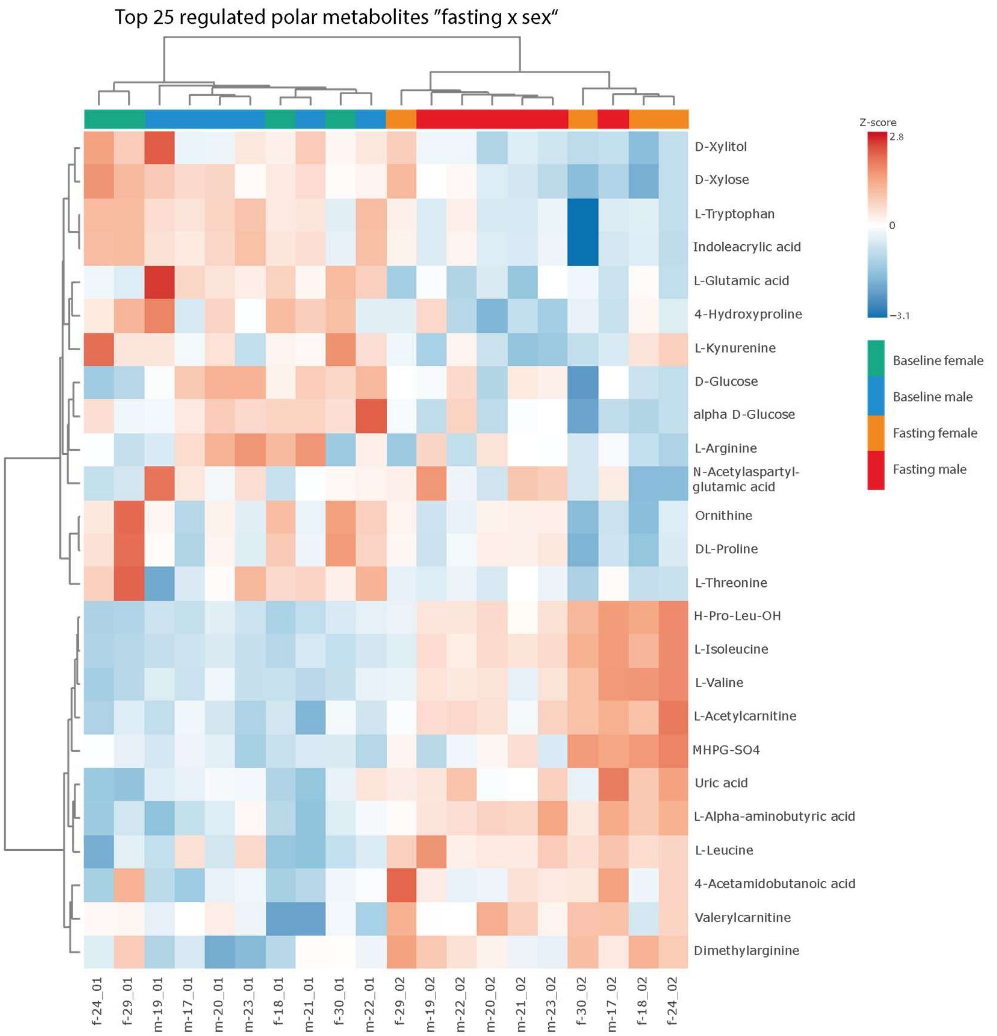

Figure S4

Heatmap of top 25 regulated polar metabolites after 72h fasting (after fasting) as compared with baseline (before fasting). Rows (samples) and columns (polar metabolites) were clustered according to Euclidean distance metrics using the Ward method. The colors show auto-scaled data of the sqrt AUCs for each metabolite. Autoscaling refers to Z-transformation to a common mean and variance of 1. Fasting and Baseline cluster in two groups. There is no consistent sex-dependent pattern.
